# The role of peer social relationships in psychological distress and quality of life among adolescents with type 1 diabetes mellitus: a longitudinal study

**DOI:** 10.1186/s12888-024-05692-5

**Published:** 2024-04-11

**Authors:** Dan Luo, Xue Cai, Hong Wang, Yubing Wang, Jingjing Xu

**Affiliations:** 1 School of Nursing, Nanjing University of Chinese Medicine, Jiangsu Province, Nanjing, China; 2 Nursing Department, Zhongda Hospital, Southeast University, Nanjing, China; 3 Department of Endocrinology, The First Affiliated Hospital with Nanjing Medical University, Nanjing, China; 4 Department of Endocrinology, Children’s Hospital of Nanjing Medical University, Nanjing, China; 5 Department of Nursing, The First Affiliated Hospital with Nanjing Medical University, Nanjing, China; 6 School of Public Health, Nanjing Medical University, Nanjing, China

**Keywords:** Coping, Diabetes distress, Pediatric diabetes, Peer, Quality of life

## Abstract

**Background:**

Adolescents with type 1 diabetes mellitus suffer from diabetes distress and poor health-related quality of life (HRQOL) since living with the condition that differentiates them from their peers. The present study investigated the effects of peer support and stress on diabetes distress and HRQOL and whether positive coping mediated the effects.

**Methods:**

We used a prospective study design. A total of 201 adolescents with type 1 diabetes mellitus from 20 cities in 4 provinces were recruited.Participants complete two separate surveys at approximately 18-month intervals. The scales employed at both Time 1 and Time 2 included the Diabetes-Specific Peer Support Measure, Diabetes Stress Questionnaire for Youths, Simplified Coping Style Questionnaire, 5-item Problem Areas in Diabetes Scale, and the Diabetes Quality of Life for Youth scale.

**Results:**

Baseline peer stress directly predicted diabetes distress and HRQOL at 18 months, even controlling for age, gender, and peer support. However, the direct effect of baseline peer support on 18-month diabetes distress and HRQOL was insignificant. Baseline peer support indirectly affected diabetes distress and HRQOL at 18 months through positive coping, indicating that positive coping plays a mediating role.

**Conclusion:**

The findings suggest that peer social relationships, especially peer stress, and positive coping are promising intervention targets for adolescents facing challenges in psychosocial adaptation.

**Supplementary Information:**

The online version contains supplementary material available at 10.1186/s12888-024-05692-5.

## Background

Type 1 diabetes mellitus(T1DM) is one of the most common incurable pediatric medical illnesses, and over 149,500 children and adolescents worldwide are diagnosed with T1DM each year [1]. More than two-thirds of children and adolescents with T1DM failed to achieve clinically recommended levels of glycemic control [2], putting many adolescents at risk for acute short-term health outcomes and long-term complications [3]. Further, during puberty, diabetes management responsibilities are transferred from parents to adolescents themselves [4]. In trying to meet the daily treatment and self-care requirements, adolescents with T1DM experience socio-psychological problems, including elevated diabetes distress [5] and impaired quality of life [6].

Diabetes distress refers to the negative emotions that arise from managing diabetes [5], encompassing feelings of fear or depression due to the condition, concerns about potential complications, as well as the daily exhaustion of excessive energy and physical strength because of diabetes. A systematic review revealed that around one-third of adolescents suffered from diabetes distress [5]. Previous studies proved that higher diabetes distress is associated with worse self-management and glycemic control outcomes among adolescents with T1DM [7, 8]. Moreover, adolescents with high diabetes distress are more likely to develop pathological forms of depression and experience a worse impact on quality of life [9, 10]. Health-related quality of life (HRQOL) is related to an individual’s well-being concerning their physical health, worries about health, and the impact of a specific health condition on physical, social, and emotional functioning [11]. HRQOL has been recognized as a critical psychosocial outcome for adolescents with T1DM. A higher level of HRQOL was associated with better glycemic control [12, 13]. Guidelines from the American Diabetes Association and the International Society of Pediatric and Adolescent Diabetes recommend that addressing diabetes distress and improving quality of life be essential to adolescents’ diabetes care [14, 15].

Peer relationships play a particularly salient role in the life of adolescents with T1DM since they become more involved in extracurricular activities that keep them away from parental supervision [16]. According to the Self-Determination Theory and its extensions [17, 18], interpersonal support is essential to meet the basic psychological human need for well-being. At the same time, problematic peer relationships may lead to loneliness, social anxiety, and depressed mood [17]. Therefore, we distinguished positive and negative peer relationships in the current study. Peer support means instrumental, emotional, or informational resources from friends or schoolmates [19]. Data from 53 countries showed a negative link between peer support and psychological distress in healthy adolescents [20]. Studies in pediatric diseases found that adolescents with higher levels of peer support enjoy a better HRQOL [21, 22]. Peer stress involves negative thoughts and reactions from friends or schoolmates or conflicts with them [23]. Previous studies revealed that higher levels of peer stress were associated with more psychopathological symptoms and poorer HRQOL among adolescents with chronic diseases and healthy peers [24, 25]. Despite the potential for peers to influence self-management and glycemic control among adolescents with T1DM [16], relatively little research has studied the influence of peer relationships on diabetes distress and HRQOL. A recent qualitative study found that adolescents with T1DM are distressed by some peers who are self-assured about the impossibility of sugar consumption in adolescents with T1DM or who deliberately compare T1DM teens to drug users [26]. A cross-sectional study found that T1DM-specific peer conflict was negatively associated with HRQOL [27].

Individuals may employ various coping strategies to maintain healthy functioning when faced with stressors [28]. Empirically, adolescents with T1DM with positive, situation-fit, and flexible coping strategies tend to exhibit less diabetes distress [29] and higher HRQOL [30]. Furthermore, positive coping strategies, such as problem-solving, emotion regulation, and cognitive restructuring, were positively associated with peer support and negatively associated with peer stress in different populations [31, 32]. The Childhood Adaptation to T1DM Model emphasizes that the interaction of peer relationships and coping styles can impact adolescents’ mental health and HRQOL [33]. Accordingly, peer support and peer stress might directly influence positive coping and might also indirectly influence subsequent diabetes distress and HRQOL through positive coping among adolescents with T1DM. Nevertheless, these hypothetical pathways have not been tested previously. McInnis and colleagues [34] found that problem-focused coping had a significant mediator role between unsupportive peer interaction and depressive symptoms among undergraduate students with polymorphism. Ji et al. [35] surveyed 221 Chinese shadow education tutors and identified the accumulative effects of social support (including peer support) and positive coping on life satisfaction. Both studies used cross-sectional designs. To prove the existence of mediating effects, the possible causes, mediators, and outcomes needed to be measured at different time points by the prospective design.

Elucidating the mechanism of preventing diabetes distress and promoting HRQOL will help healthcare workers design comprehensive interventions to improve mental health and well-being in adolescents with T1DM.However, there is currently no research that simultaneously investigates the effect of peer relationships and positive coping on diabetes distress or HRQOL in adolescents with T1DM. Thus, this study expands on the existing literature by investigating a mediation paradigm between peer relationships and positive coping with diabetes distress and HRQOL. Precisely, we hypothesized that (1) peer support would directly affect diabetes distress and HRQOL; (2) peer stress would directly affect diabetes distress and HRQOL; (3) positive coping would mediate the relation that peer support has with diabetes distress and HRQOL; (4) positive coping would mediate the relation that peer stress has with diabetes distress and HRQOL.

## Methods

### Study design

The current study is longitudinal, with an 18-month follow-up, which started on June 10, 2020. Data were collected in two waves.

### Participants and settings

Participants were consecutively recruited from the First Affiliated Hospital with Nanjing Medical University and the Children’s Hospital of Nanjing Medical Universityin China. Inclusion criteria were: (i) being diagnosed with Type 1 diabetes for more than six months, (ii) age between 10 and 19 years, (iii) being fluent in oral Mandarin, and (iv) being able to understand and complete the questionnaire. We excluded participants if they suffered frommajor psychiatric disorders (e.g.,schizophrenia,major depressive disorder, bipolar disorder, substance use disorder), neurocognitive disorder (e.g., delirium and dementia), or diabetes complications during recruitment. The G.power 3.1.7 was used to calculate the sample size. The regressions we performed to examine the impact of peer relationships on diabetes distress and HRQOLincluded a maximum of six predictors: Time 1 and Time 2 peer support, Time 1 and Time 2 peer stress, and participants’ age and gender. We chose an Alpha of 0.05. Power (1-β) was set to 0.95. The effect size f^2^ was small (0.1) to ensure a sufficient sample size. Therefore, a 132-subject sample size was estimated to be necessary. All procedures performed in this study are under the Declaration of Helsinki. We collected data after approval by the Bioethics Review Committee of the Peking University Health Science Center(numberIRB00001052-19108).

### Data collection

**Fig. 1:**
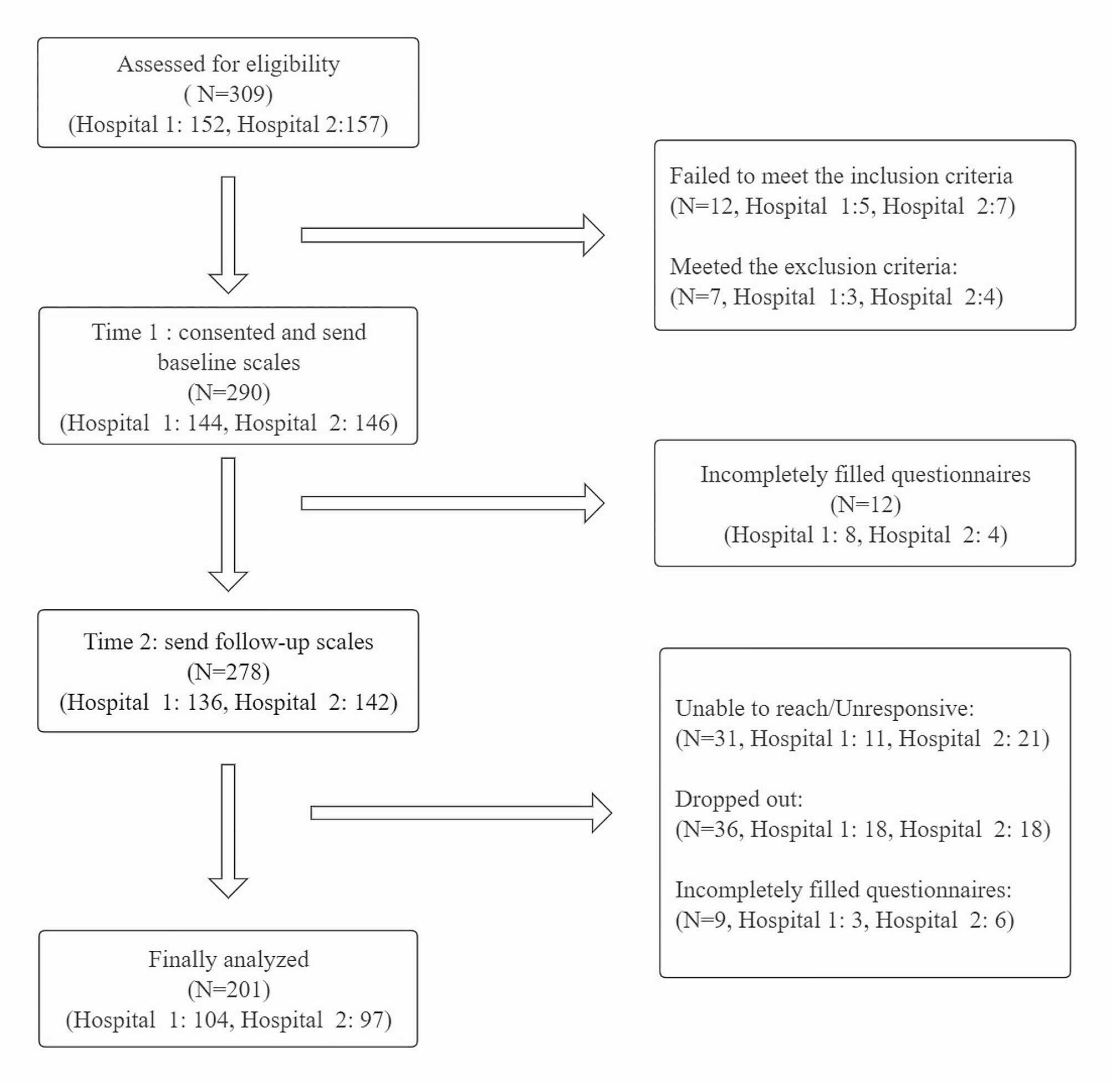
Participant recruitment flow chart

Two-wave studies have been completed with twelve-time intervals, designated as Time 1 (June 10, 2020 to August 20, 2021) and Time 2 (December20, 2021 to February 15, 2023).A diabetes specialist nurse contacted all adolescents with Type 1 diabetes who visited the endocrinology clinic and asked them if they would be interested in participating in the study. Informed consent to participate in the study was obtained from participants. Parents of participants under legal age (< 16 years) provide written informed consent before inclusion into the study. Fig.1 shows the detailed patient recruitment process. Adolescents underwent structured pen-and-paper surveys in a meeting room without the presence of their guardians. At time 1, 290 participants from two hospitals in China completed the initial survey. After completing the baseline questionnaires, adolescents were required to come to the hospital for a follow-up assessment at 18 months. At Time 2, the participants completed the same questionnaires, albeit with a slight change in the order, aiming to encourage conscientious completion. Participants will be contacted by phone and receive a reminder email two weeks before theirfollow-up date.

### Measures

Anonymous self-reported questionnaires collected demographic characteristics, peer support, peer stress, positive coping, diabetes distress, and HRQOL. The various measures reported in this publication are described in the SupplementalMaterial.Disease information and HbA1c levels were obtained from medical records.

*Peer support.* Six items evaluated participants’perceptions of diabetes-specific support received from friends.Items 1 to 3 were derived from the emotional support subscale of theDiabetes Social Support Questionnaire–Friends [36] to assess the degree offriends’ understanding and encouragement regarding diabetes. Participants rated each item for frequency (1 = never, 5 = at least once daily).Items 4 to 6 were derived from Pihlaskari’s study to capture how peers could assist in managing T1DM [19]. Each item was scored from 1(strongly disagree) to 5(strongly agree). We added the scores of the six items to form a single diabetes-specific peer support score. The overall score ranged from 6 to 30,with higher scores indicating higher levels of peer support. The Chinese version of DSPSM in this sample had acceptable internal consistency (Cronbach’s α = 0.85), test-retest reliability (intraclass correlation coefficient = 0.89), and construct validity (one factor explained 64.23% variance).

*Peer stress.* The peer stress subscale of the Diabetes Stress Questionnaire for Youths (DSQY) [23] was used to measure peer-related diabetes stress. The peer stress subscale comprised eight items, including telling peers about having diabetes and effectively managing it in their presence or experiencing hypoglycemia during social gatherings with peers. All items were rated on a 4-point Likert scale (0 = not at all, 3 = very much). The overall score ranged from 0 to 24, with higher scores indicating more peer stress. In this sample, the Cronbach’s α of the Chinese versions of the peer stress subscale was 0.92, and the intraclass correlation coefficient was 0.87. The exploratory factor analysis extracted one factor, explained 63.79% of the variance.

*Positive coping.* The Simplified Coping Style Questionnaire (SCSQ) [37] comprises three subscales: self-regulation (eight items), help-seeking and problem-solving (five items), and fantasy and escapism (seven items).We measured participants’ positive coping using the self-regulation and help-seeking and problem-solvingsubscales from the SCSQ. Each item was rated from 0 (not used) to 3 (always using).The overall scoreranged from 0 to 39, with a higher score indicating a more positive coping style. The SCSQ showed good test-retest reliability and construct validity. In this sample, Cronbach’s αfor the self-regulation and help-seeking and problem-solving subscaleswas 0.88.

*Diabetes distress.* The 5-item Problem Areas in Diabetes Scale(PAID-5) [38]measures diabetes-related psychological distress.All items were scored from 0 (not a problem) to 4 (a severe problem). The overall score ranged from 0 to 20, with higher scores reflecting more severe distress. The PAID-5 is reliable and valid, widely used in mainland China and adolescents with Type 1 diabetes [39]. The Cronbach’s α in this sample was0.83.

*Health-related quality of life.* HRQOL was evaluated by the life satisfaction and diabetes impact subscales of the Chinese Diabetes Quality of Life for Youth scale (C-DQOLY-SF) [40]. The life satisfaction subscale consisted of eight items that measured adolescents’ satisfaction with treatment and school life. Each item was rated from 1 (very unsatisfied) to 5 (very satisfied).The diabetes impact subscale contained nine items that assessed diabetes-related symptoms, impact on activities, and parent concern. Each item was rated from 1 (never) to 5 (all the time). The overall score of the life satisfaction and diabetes impactsubscales ranged from 17 to 85, with higher scores reflecting better HRQOL. The reported reliability and validity of the C-DQOLY-SF were promising [40]. In this sample, Cronbach’s α of the life satisfaction and diabetes impact subscaleswas 0.93.

### Statistical analysis

First, descriptive analysis was conducted using SPSS 22.0 to explore the demographic and disease characteristics of the participants.Second, the independent samples tests were applied to analyze the significant differences between study variables across study waves. Third, multivariate linear regression models were built to examine whether Time 1 peer relationships predict diabetes distress and HRQOL at Time 2 when controlling for the effects of peer relationshipsat Time 2. Participants’ age and gender, which demonstrated a correlation with diabetes distress and HRQOL in previous studies [41, 42], were entered into models as covariates.

Subsequently, we employed Mplus 7.4 statistical software to test the mediating effects of positive coping using the half-longitudinal mediation model, applying maximum likelihood estimation with robust standard errors (MLR). The semi-longitudinal mediation model’s fit and credibility may be compromised when integrating two dependent variables within the same model [43]. Given that previous studies have already demonstrated a negative association between diabetes distress and HRQOL [44], we analyzed diabetes distress and HRQOL using separate models. According to Cole and Maxwell’s recommendation regarding two waves of longitudinal studies [43], we calculated the product of the regression coefficient from Time 1 peer relationships to Time 2 coping and the regression coefficient from Time 1 coping to both diabetes distress and HRQOL at Time 2, respectively. The regression coefficients were utilized in the Sobel test to assess the indirect effects of peer relationships on diabetes distress and HRQOL. When the *P*-value for the Sobel test is < 0.05, the mediating effect of positive coping is statistically significant. The following criteria were used to appraise the model fit [45]: χ^2^/DF ≤ 5.00, comparative fit index (CFI) ≥ 0.90, the goodness of fit index (GFI), incremental fit index (IFI) ≥ 0.90, and standardized root mean square residual (SRMR) ≤ 0.08.

## Results

### Characteristics of participants and levels of measured variables across study waves

A response rate of 90% to the first surveyand 69% to the follow-up survey yielded 201 participants with complete responses to two times questionnaires. The present study observed no significant differences (all *P* > 0.05) in all demographics and disease variables between the valid and missing track samples. At the baseline survey, adolescents’ mean age was 14 ± 4.36 (mean ± standard deviation) years, and 46.8% were boys. Nearly half of the participants (44.8%) lived in the urban area, and 116 (57.7%) had a monthly household income of $ 700 or more. Over half (51.2%) of the adolescents reported their parents having more than one child. Regarding the insulin treatment regimen, 67.7% of adolescents used insulin pens. The average daily total insulin dosage was 35.71 ± 16.93 IU. The mean duration time since diagnosis was 4.36 ± 3.35 years. Participants’ mean HbA1c was 7.79 ± 1.82%, and nearly half of the adolescents (49.8%) failed to reach the glycaemic goal (HbA1c < 7.5%).

The means and standard deviations of the five critical variables at Time 1 and Time 2 are shown in Table 1. According to the instrument scores, participants’ peer support (15.92 ± 6.00), peer stress (7.35 ± 5.88), and diabetes distress (7.04 ± 4.71) at baseline were below the medium levels, while baseline positive coping (26.43 ± 7.97) and HRQOL (63.27 ± 10.63) were just above the medium levels. There were nosignificantdifferences in peer support (t = -0.183, *P* = 0.866), peer stress (t = -0.169, *P* = 0.855), positive coping (t = 0.742, *P* = 0.459), diabetes distress (t = -0.183, *P* = 0.855), or HRQOL (t = 1.488, *P* = 0.138) from baseline to 18-month follow-up.

**Table 1:** Participants characteristics and levels of measured variables

Variables	Mean (SD)/N (%)
**Age, year**	14 (4.36)
**Gender**	
Boy	94 (46.8)
Girl	107 (53.2)
**Residence**	
Urban area	90 (44.8)
Rural area	113 (55.2)
**Monthly family income**, $	
< 700	85 (42.3)
≥700	116 (57.7)
**Single-child family**	
Yes	98 (48.8)
No	103 (51.2)
**Therapy method**	
Insulin pen	136 (67.7)
Insulin pump	65 (32.3)
**Diabetes duration, year**	4.36 (3.35)
**Daily total insulin dosage, IU**	35.71 (16.93)
**HbA1c, %**	7.79 (1.82)
**Peer support**	
Time 1	15.92 (6.00)
Time 2	16.01 (5.17)
**Peer stress**	
Time 1	7.35 (5.88)
Time 2	7.45 (5.57)
**Positive coping**	
Time 1	26.43 (7.97)
Time 2	27.00 (7.34)
**Diabetes distress**	
Time 1	7.04 (4.71)
Time 2	7.04 (4.49)
**Health-related quality of life**	
Time 1	63.27 (10.63)
Time 2	61.50 (13.05)

*Note* HbA1c,Glycated hemoglobin

### Effect of peer relationships on diabetes distress and health-related quality of life

The results of the multipleregressions are summarized in Table 2. All variables collectivelyexplained 18% in diabetes distress and 12% of the variance in HRQOL at Time 2, respectively.

#### Diabetes distress

After adjusting for age, gender, and peer support at Time 1 and Time 2, it was observed that peer stress at both Time 1 and Time 2 positively predicted Time 2 diabetes distress (*P* = 0.009 and 0.003, respectively). The result suggests that adolescents who perceive higher peer stress are likely to experience increased levels of diabetes distress. However, we did not observe an effect of Time 1 peer support or Time 2 peer support on Time 2 diabetes distress (*P* = 0.752 and 0.498, respectively).

#### Health-related quality of life

Time 1 peer stress negatively predicted Time 2 HRQOL (*β*= −0.46, t = -2.62, *P* = 0.010) after controlling for effects of age, gender, Time 2 peer stress, and peer support at Time 1 and Time 2. Although the Time 2 peer support is positively associated with Time 2 HRQOL (*β* = 0.52, t = 2.82, *P* = 0.005), the predictive effect of Time 1 peer support on Time 2 HRQOL is not statistically significant (*P* = 0.530).

**Table 2:** Multivariate regression model on the effects of peer relationships on diabetes distress and health-related quality of life

Predictors	Model 1 (Diabetes Distress)			Model 2 (HRQOL)		
	*β*	t	*P*	*β*	t	*P*
Age	-0.03	-0.61	0.545	-0.75	-3.14	0.002
Gender	1.55	2.21	0.028	-2.21	-1.24	0.217
T1 peer support	-0.03	-0.32	0.752	-0.11	-0.63	0.530
T1 peer stress	0.13	2.64	0.009	-0.46	-2.62	0.010
T2 peer support	0.04	-0.68	0.498	0.52	2.82	0.005
T2 peer stress	0.17	2.96	0.003	0.13	0.71	0.478

*Note* HRQOL, health-related quality of life; R^2^ for Model 1 = 0.18; R^2^for Model 2 = 0.12

### Mediation effects of positive coping on relationships between peer relationships and outcomes

#### Diabetes distress

**Fig. 2:**
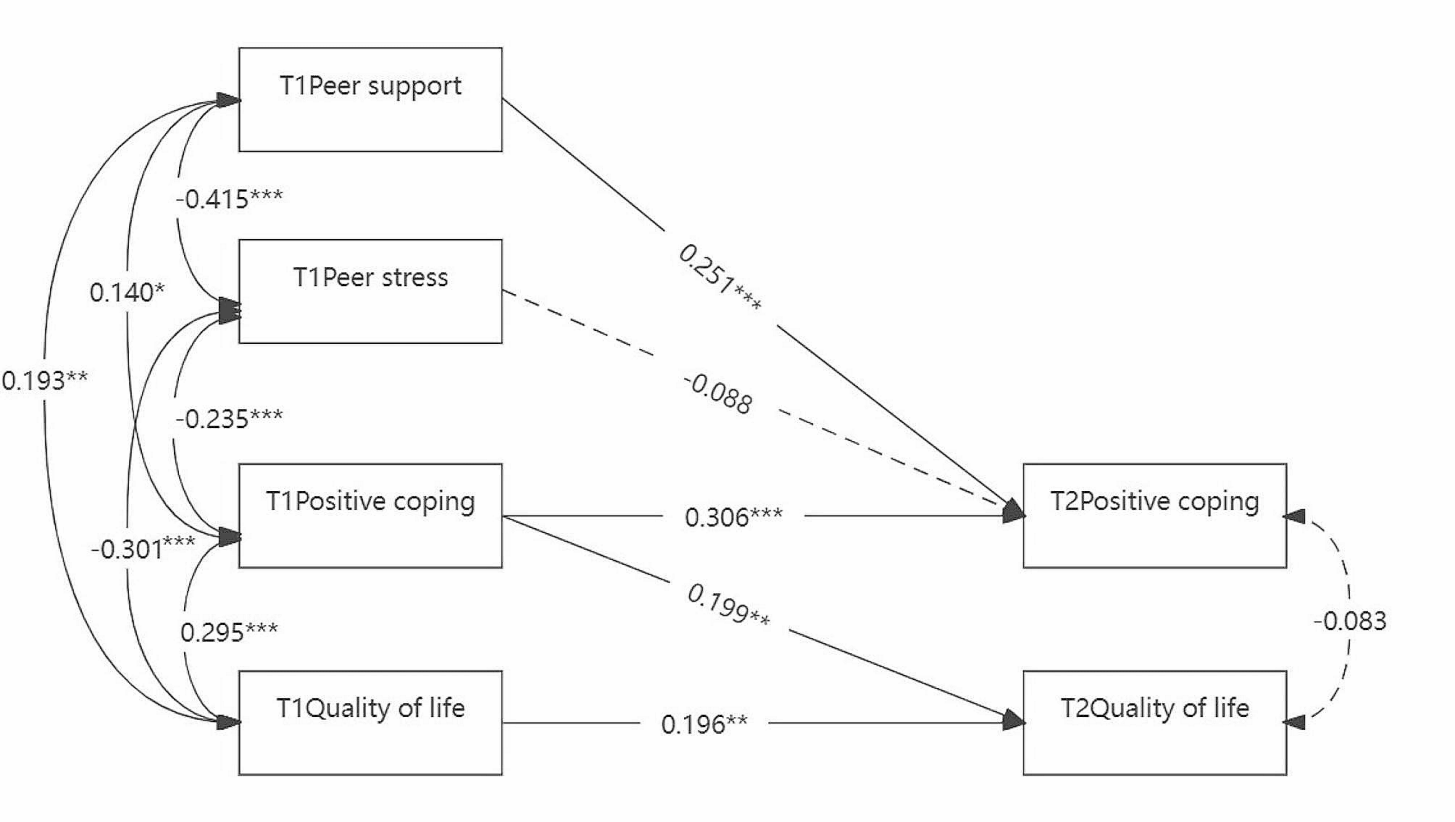
Standardized coefficients in the half-longitudinal mediation model forpeer relationships, positive coping, and diabetes distress at two-time points. Dotted lines represent insignificant paths,^*^P < 0.05, ^**^P < 0.01, ^***^P < 0.001

The half-longitudinal mediation model for diabetes distress (Fig. 2) presented an acceptable fit to the data(χ^2^/DF = 0.556, CFI = 1.000, TLI = 1.066, SRMR = 0.017).

In the model, Time 1 peer support predicted Time 2 positive coping (*β* = 0.260, *P* < 0.001), accounting for Time 1 positive coping. Furthermore, Time 1 positive coping predicted Time 2 diabetes distress (*β*= -0.228, *P* = 0.001), adjusting for the diabetes distress at Time 1. The Sobel test highlighted a significant indirect effect of peer support at Time 1 on diabetes distress at Time 2 through positive coping (t = -2.26, *P* = 0.024), indicating that positive coping was a mediator in the relationship between peer support and diabetes distress.

We failed to find a significant effect of Time 1 peer stress on Time 2 positive coping (*β*= -0.080, *P* = 0.247). The subsequent Sobel test suggested that peer stress did not affect diabetes distress indirectly (t = 1.070, *P* = 0.285).

### Health-related quality of life

**Fig. 3:**
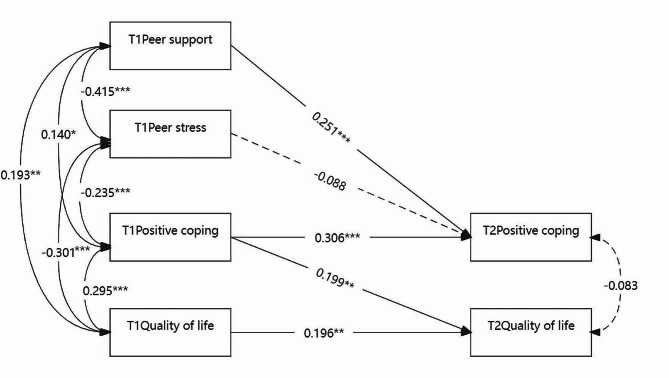
Standardized coefficients in the half-longitudinal mediation model for peer relationships, positive coping, and health-related quality of life at two-time points. Dotted lines represent insignificant paths,^*^P < 0.05, ^**^P < 0.01, ^***^P < 0.001

The results showed that the half-longitudinal mediation model for HRQOL (Fig. 3) has a fit index (χ^2^/DF = 1.645, CFI = 0.971, TLI = 0.913, SRMR = 0.024). The model revealed that peer support at Time 1 predicted positive coping at Time 2 (*β* = 0.251, *P* < 0.001) while controlling for Time 1 positive coping. Additionally, Time 1 positive coping predicted Time 2 HRQOL (*β* = 0.199, *P* = 0.004), adjusting the baseline HRQOL. The Sobel test indicated a significant indirect effect of Time 1 peer support on Time 2 HRQOL through positive coping (t = 2.25, *P* = 0.025). Positive coping mediated the relationship between peer support and HRQOL.

However, the predictive effect of Time 1 peer stress on Time 2 positive coping is not statistically significant (*β*= -0.088, *P* = 0.209). Further, the Sobel test indicated that peer stress did not indirectly affect HRQOL (t = -1.14, *P* = 0.252).

## Discussion

During adolescence, a re-orientation of the social network occurs, in which peers are favored. Although the potential for peers to influence self-management and glycemic control has been discussed in adolescents with T1DM, the effects of peer relationships on diabetes distress and HRQOL are yet to be fully understood. First, we found that.

Baseline peer stress directly predicted diabetes distress and HRQOL at 18 months,while the direct effect of baseline peer support on 18-month diabetes distress and HRQOL was insignificant. Second, positive coping mediated the effects of peer support on diabetes distress and HRQOL. The implications of these findings provide a greater understanding of a mechanism that may explain how peer relationships influence diabetes distress and HRQOL in adolescents with T1DM.

This study was the first to report that higher levels of baseline peer stress directly resulted in less 18-month diabetes distress among adolescents with T1DM. This finding was consistent with a longitudinal study that reported that more chronic experiences of peer stress predicted more distress and anxiety in Asian American 9th–10th graders [46]. Moreover, we found that baseline peer stress directly affects 18-month HRQOL, similar to a population-based study of children with psoriasis [47]. The above findings further confirmed that peer stress is a crucial stressor influencing subsequent mental health and well-being among adolescents with T1DM. Because discrimination, rejection, and unfavorable judgment from peers lead to stigma and poor self-management [48], resulting in diabetes distress and worse HRQOL [49, 50]. In this prospective study, baseline peer support did not directly affect diabetes distress and HRQOL at 18 months. A recent systematic review also concluded that technology-based peer support interventions for chronically ill adolescents had mixed effects on HRQOL [51]. However, Raymaekers et al. [52] followed 467 late adolescents and emerging adults with Type 1 diabetes in Belgium and found that peer support negatively predicted diabetes-related distress over time. Differences in the age and country of the participants in the two studies may have contributed to the inconsistent result.Peers’ support may be erroneous, and adolescents recognize some supportive behaviors with T1DM as nagging [53]. Sometimes, diabetes-specific peer support may threaten the adolescent’s self-concept from a ‘normal’ adolescent towards a sick role [53]. Combining the above findings, healthcare workers had a better focus on identifying and removing peer stress and providing peers with knowledge regarding diabetes care and emotional support skills.

Our results extend previous study findings by providing a more nuanced perspective of the relations among peer relationships, positive coping, diabetes distress, and HRQOL. We found that positive coping mediated the relationship between baseline peer support and 18-month diabetes distress. This result suggested that higher levels of peer support led to more positive coping behaviors, which contributed to decreased diabetes distress among adolescents with T1DM. Cheng et al. [54] examined the effectiveness of the peer-led intervention, including coping training, for people with Type 2 diabetes and reported significant reductions in diabetes distress three months after the intervention. Furthermore, we found that positive coping mediated the relationship between baseline peer support and 18-month HRQOL. Adolescents with T1DM who respond positively are more inclined to disclose their diabetes condition to peers and seek support from parents and peers [55], thereby contributing to an improved HRQOL. Our study confirmed specific paths in the Childhood Adaptation to T1DM Model [33]. According to this model, social competence and coping collaborate to predict psychosocial responses and adaptation. Diabetes distress is categorized as a psychosocial response, while quality of life represents the level of adaptation.Contrary to our research hypothesis, peer stress did not have indirect effects on both diabetes distress and HRQOL through positive coping. Research has demonstrated that adolescents’ coping responses to peer stress can affect their subsequent moods and well-being [56]. Santiago et al. found that engagement coping buffered the effect of poverty stress on next-day negative mood among 58 Latino adolescents [57]. Hence, positive coping may serve as a moderating factor rather than a mediating one in the relationship between peer stress and both diabetes distress and HRQOL. Further research is necessary to validate this hypothesis.

In clinical care, we recommend integrating assessments of peer relationships and coping styles as part of routine evaluations for adolescents’ psychosocial functioning. Standard screening in diabetes clinics may help adolescents utilize peer resources and identify those who need further help to address peer stress. Healthcare workers should provide psychological counseling and coping skill training for adolescents with T1DM who are faced with significant peer stress so that they can learn methods to respond more positively and deal with the adverse effects. In addition, coping-based intervention strategies, such as analysis of the problem, defining goals, and modification of coping, should be included in a peer-led support program to help adolescents process diabetes distress.

Conclusions based on the current results should be made cautiously considering the following limitations. First, we only conducted two waves of investigations. Although data from two waves can also be used to test the mediation effect [43], future studies should examine the continuoustime relationships between the study variables using three-wave surveys. Second, the generalizability of the results might be limited, as we enrolled participants in two hospitals in China. Third, we investigated only positive coping. Negative coping strategies like fantasy, escape, overeating, and self-harm may also be involved in the associations between peer relationships, diabetes distress, and HRQOL. Moderation analyses need to be conducted to understand the relationships between peer variables, different coping styles, and psychosocial health in adolescents with T1DM. Finally, variables in this study were measured by self-reported instruments, which might lead to shared method variance and social desirability response. Collecting data from the perspective of both adolescents and peers is wise.

## Conclusion

In conclusion, the present study is the first to examine the influential pathways from peer relationships to diabetes distress and HRQOL by prospective design. Baseline peer stress directly predicted 18-month diabetes distress and HRQOL, even controlling for age, gender, and peer support. Baseline peer support indirectly affected 18-month diabetes distress and HRQOLthrough positive coping. The findings of this study indicated that peer relationships,especially peer stress, and positive coping should be simultaneously considered to relieve diabetes distress and improve HRQOL in adolescents with T1DM.

### Electronic supplementary material

Below is the link to the electronic supplementary material.

Supplementary Material 1

## Data Availability

The datasets used and/or analysed during the current study are available from the corresponding author on reasonable request.

## Abbreviations

T1DM: Type 1 Diabetes Mellitus
HRQOL: Health-Related Quality of Life
DSPSM: Diabetes-Specific Peer Support Measure
DSQY: Diabetes Stress Questionnaire for Youths
SCSQ: Simplified Coping Style Questionnaire
PAID-5 5-item: Problem Areas in Diabetes Scale
C- DQOLY-SF: D- Chinese version Diabetes Quality of Life for Youth scale
CFI: Comparative Fit Index
GFI: Goodness of Fit Index
IFI: Incremental Fit Index
SRMR: Standardized Root Mean Square Residual
