# Supplementary material for: The role of peer social relationships in psychological distress and quality of life among adolescents with type 1 diabetes mellitus: a longitudinal study
Source: BMC Psychiatry. 2024 Apr 11;24:270. doi: 10.1186/s12888-024-05692-5 (PMC11010305; doi:10.1186/s12888-024-05692-5)
Supplement: Supplementary file 1 — Supplementary Material 1 [file 12888_2024_5692_MOESM1_ESM.docx]

**The Scales used to measure study variables**

The instructions and items used to measure peer support, peer stress, positive coping, diabetes distress, and health-related quality of life were described as follows.

**Peer support**

***Items 1 to 3***

Kindly rate items 1 to 3 based on the frequency of the behavior exhibited by the friends with whom you spend the most time (1 = never; 2 = less than twice a month; 3 = once a week; 4 = several times a week; 5 = at least once a day).

1. Encourage you to do a good job of taking care of your diabetes

2. Understand when you sometimes make mistakes in taking care of your diabetes

3. Are available to listen to concerns or worries about your diabetes care

***Items 4 to 6***

Please indicate the extent to which you agree or disagree with the following statements about your friends and your diabetes (1 = strongly disagree; 2 = disagree; 3 = somewhat disagree and agree; 4 = agree; 5 = strongly agree).

4. The friends I currently spend the most time with know I have diabetes

5. These friends are helpful in providing support for my diabetes

6. These friends know what to do when I have an emergency with my diabetes (e.g., have an insulin reaction, go low, etc.)

**Peer stress**

Please read the following situations and rate how stressful they are; in other words, rate how upsetting, difficult, or how much of a problem each one is for you by circling one of the following: Not at all; A little; Pretty much; Very much. There are no right or wrong answers, only what is true for you. Think about each situation and then rate how stressful or how much of a hassle it is for you personally.

1. Test blood with friends

2. Take shot with friends

3. Wear ID bracelet

4. Kids ask about diabetes

5. Tell about diabetes

6. Tell kids about diabetes

7. Talk with friends about diabetes

8. Treat reaction when with friends

**Positive coping**

The following sentences describe attitudes and methods that you may adopt when facing setbacks. Please choose a number to indicate the frequency with which you employ the following coping strategies (1 = never; 2 = sometimes; 3 = often; 4 = always). Note that there are no right or wrong answers.

***Self-regulation subscale***

1. Find relief through work, study, or other activities
2. Strive to see the positive side of things
3. Adjust your perspective, rediscovering what is more important in life
4. Avoid taking problems too seriously
5. Stand firm in your position and strive for what you desire
6. Explore hobbies and participate in activities you enjoy
7. Exercise emotional restraint, especially in moments of disappointment, regret, sadness, or anger
8. Temporarily set aside problems or worries

***Help-seeking & problem-solving subscale***

1. Engage in conversations and confide inner troubles with others.
2. Seek solutions to problems
3. Seek advice from others
4. Modify your approach
5. Draw from others’ experiences

**Diabetes distress**

Which of the following diabetes issues are currently a problem for you? Circle the number that gives the best answer for you. Please provide an answer for each question (0 = Not a problem; 1 = Minor problem; 2 = Moderate problem; 3 = Somewhat serious problem; 4 = Serious problem).

1. Feeling scared when you think about living with diabetes
2. Feeling depressed when you think about living with diabetes
3. Worrying about the future and the possibility of serious complications
4. Feeling that diabetes is taking up too much of your mental and physical energy every day
5. Coping with complications of diabetes

**Health-related quality of life**
The following questions are designed to investigate the diabetes quality of life, including both life satisfaction and the impact of diabetes. Please carefully read the following sentence and select the option that best fits your actual situation. For life satisfaction subscale, 1= never; 2 = very seldom; 3 = sometimes; 4 = often; 5 = all the time. For Diabetes impact subscale, 1= very unsatisfied; 2 = somewhat unsatisfied; 3 = neither; 4 = somewhat satisfied; 5 = very satisfied.

***[life satisfaction](javascript:;) subscale***

1. How satisfied are you with the amount of time it takes to manage your diabetes
2. How satisfied are you with the amount of time you spend getting checkups
3. How satisfied are you with the amount of time it takes to determine your sugar level
4. How satisfied are you with your current treatment
5. How satisfied are you with the flexibility you have in your diet
6. How satisfied are you with your performance in school
7. How satisfied are you with how your classmate treat you
8. How satisfied are you with your attendance at school

***Diabetes impact subscale***

1. How often do you feel pain associated with the treatment for your diabetes
2. How often do you feel physically ill
3. How often does your diabetes interfere with your family life
4. How often do you have a bad night’s sleep
5. How often does your diabetes interfere with your exercising
6. How often do you find that your diabetes interrupts your leisure-time activities
7. How often do you find that your parents are too protective of you
8. How often do you feel that your parents worry too much about your diabetes
9. How often do you find that your parents act like diabetes is their disease, not yours
